# Melatonin, Zinc, and Vitamin C: Potential Adjuvant Treatment for COVID-19 Patients

**DOI:** 10.3389/fnut.2021.821824

**Published:** 2022-01-26

**Authors:** Leandro Borges, Matheus Gennari-Felipe, Beatriz Belmiro Dias, Elaine Hatanaka

**Affiliations:** Interdisciplinary Program of Health Sciences, Cruzeiro Do Sul University, São Paulo, Brazil

**Keywords:** SARS-CoV-2, clinical trial, observational study, therapy, hyperinflammation

## Abstract

The use of nutraceutical approaches may regulate the immune system, performing a potential strategy to contain the worst outcomes of COVID-19. We reviewed the current evidence surrounding nutritional/nutraceutical approaches for the therapy in patients with COVID-19. We searched the PubMed database to report randomized controlled trials (RCTs) and observational research that used melatonin, zinc, or vitamin C supplementation as an intervention for COVID-19 treatment. To date, we found only three concluded studies that assessed zinc supplementation and melatonin therapy in patients with COVID-19, but with inconclusive data, relatively small sample size, and early termination of the trial. On the other hand, vitamin C therapy appears to reduce hyperinflammation and improve the oxygen support status of patients with COVID-19. However, a large part of this research involves pilot trials, and there are still conflicting data regarding mortality rate, mechanical ventilation, and duration of symptoms of patients with COVID-19. Melatonin, zinc, and vitamin C supplementation should be investigated further on the nutritional status-immune response, and data from ongoing trials are needed to understand these molecules as a therapy strategy in patients COVID-19.

## Introduction

Coronaviruses are a family of viruses that induce intestinal and respiratory disorders in animals and humans. They generally induce mild colds in the population, but the arising of the severe acute respiratory syndrome (SARS) epidemic (2002–2003) as well as the Middle East respiratory syndrome (MERS) (2012) demonstrated that they can also induce serious illness (1). The severe acute respiratory syndrome coronavirus 2 (SARS-CoV-2) has confirmed diagnoses that presently exceeded 274,628,461 people worldwide and nearly 5,358,978 deaths (2). Although pharmaceutical trials are focused on new drugs for COVID-19, due to frequently occurring virus mutations and drug ineffectiveness (3), these studies are time-consuming and inconclusive. Determining a drug treatment is of utmost importance since distinct drugs may be efficient at particular stages of viral infection. For instance, adjunct approaches such as immunomodulators may be functional at an early phase of the infection, while antiviral drugs (e.g., remdesivir) can be more efficient for severe patients with COVID-19. Moreover, toxicity dose selection, as well as no side-effect, are critical components that are related to the efficacy and safety of the drug (4). In this scenario, many trials are assessing the success of safe and cheap nutritional/nutraceutical approaches targeting immune regulatory pathways, viral proteins, or the viral entry pathway, and the “nutritional status–immune response” dyad of a person becomes even more relevant during the COVID-19 pandemic (4, 5).

Some molecules and nutrients play central roles in keeping the function and integrity of the immune system, showing synergistic results in steps that are crucial for the immune response. Among these elements, melatonin, zinc, and vitamin C present robust evidence of their immunomodulating activity, such that their deficit, even if superficial, can harm the metabolism and, hence, their response on the immune system (6, 7). Additional discussion on this topic can be explored through the idea grounded by the Law of the Minimum suggested by Justus von Liebig (8) and the triage hypothesis suggested by Ames (9).

Viral infections elevate the necessity for vitamins, such as A, B, C, and D (10). Notwithstanding the absence of preclinical data of effectiveness against coronaviruses, ascorbic acid (vitamin C), a water-soluble nutrient that has antioxidant potential, is a broadly used supplement nowadays (11). In humans, preclinical trials indicate that vitamin C ameliorates immunoregulation and the outcomes of inflammation by preventing proinflammatory cytokine release, controlling reactive oxygen species, and defending host cells (12, 13). These effects, associated with low toxicity, motivated recent research to add high-dose intravenous vitamin C (HDIVC) to the regular therapy of patients with severe illnesses, such as acute respiratory distress syndrome (ARDS) (14), sepsis (14, 15), and cardiac surgery (16).

Zinc gluconate is a generally accessible over-the-counter supplement that people use for viral illnesses treatment. Zinc regulates the immune response *via* white blood cell and antibody production (17). Recent studies indicated that angiotensin-converting enzyme (ACE)-2 expression is modulated by Sirtuin 1 (SIRT1) and that zinc reduces SIRT1 response; therefore, the modulation of SIRT1 by zinc could reduce ACE-2 expression and lastly viral entrance into the cell (18, 19). Research in cell culture also found that zinc can restrict the RNA polymerase of SARS-CoV-1 (20), and the proteolytic processing of polyproteins in RNA viruses (21). Moreover, zinc deficiency reduces the production of antibodies and elevates proinflammatory biomarkers (22). Most relevant is the fact that continuous low serum zinc has been inversely correlated with mortality from sepsis and related to recurrent sepsis (23, 24), highlighting the value of the zinc approach in COVID-19 treatment.

Melatonin (*N*-acetyl-5-methoxytryptamine) is known to promote antioxidative, antiinflammatory, and immunomodulation effects (25, 26), and melatonin exposure postintubation is related to the positive result in patients with COVID-19 (27). Since melatonin is a small molecule with amphiphilic essence and can spontaneously come into all cells and quickly go through biological membranes to achieve subcellular structures and organelles (28), this indoleamine has been hypothesized to be efficient to restrain viral infections by the interaction with the coronavirus membrane and its genetic component. The SARS-CoV-2 virus stimulates the nod-like receptor family, pyrin domain-containing three inflammasome (29), activating nuclear factor kappa-B (NF-κB) and resulting in cytokines expression and release (30), and leukocyte dysfunction (31). Besides, melatonin restores the lungs from oxidative injury induced by age (32), an effect that may be significant to decrease the inflammatory damage and local oxidative in the lungs of patients with COVID-19.

**Figure 1:**
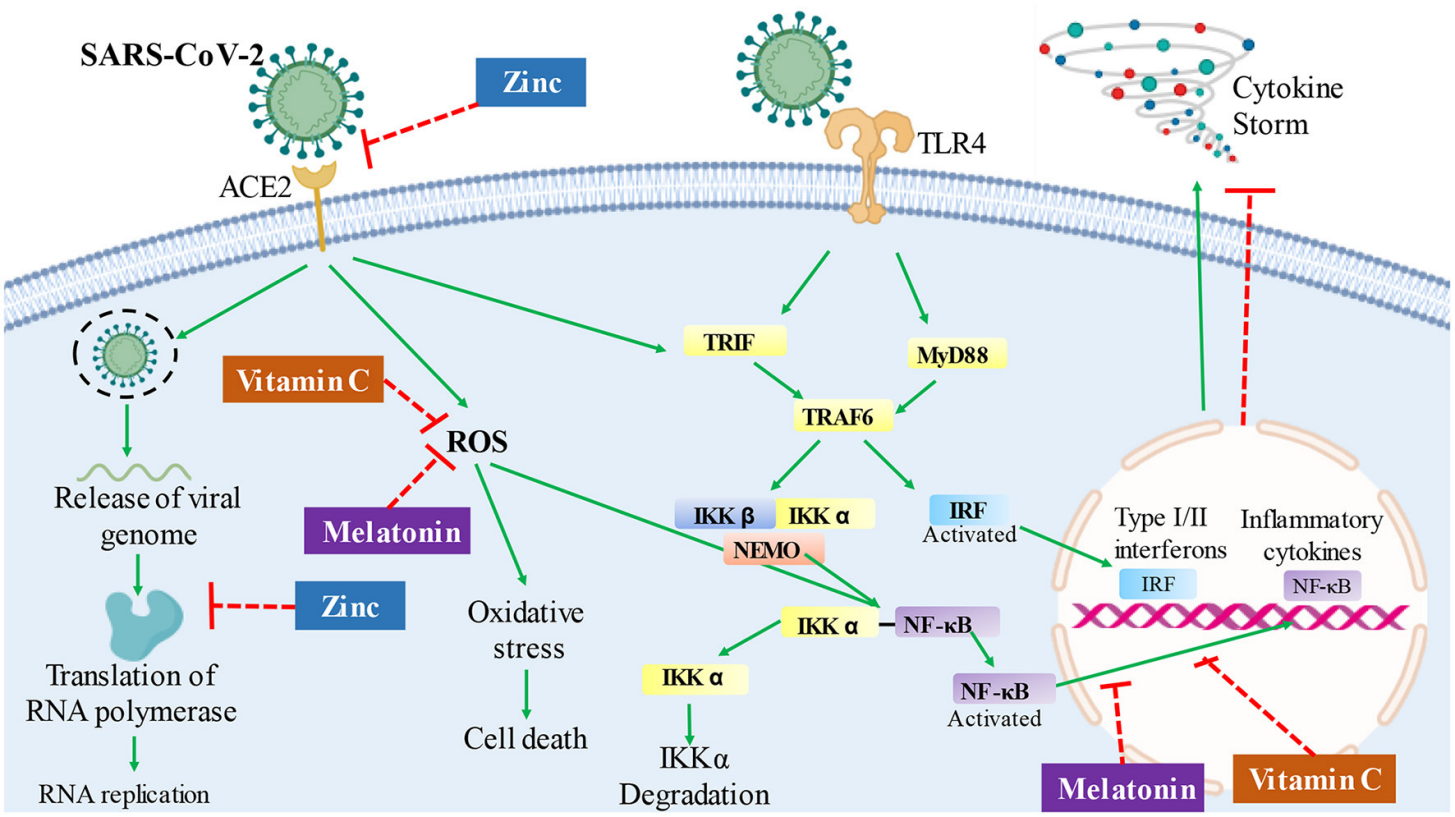
Hypothesis of potential routes and mechanisms, in which melatonin, zinc, or vitamin C therapy could affect the infection response in patients with COVID-19. The overreactive immune system response, followed by severe inflammation and oxidative stress, can contribute to COVID-19 pathology, leading to a cytokine storm. Vitamin C has an antioxidant activity, which could prevent oxidative stress and regulate cytokine production by deactivating the NF-kB signaling cascade in patients with COVID-19. Melatonin also has antioxidant activity and decreases NF-kB activation, which could contribute to the inhibition of cytokine storm. Besides, zinc could act by decreasing viral replication by inhibiting RNA polymerase and reverse transcriptase activity; in addition, it could decrease the expression of the ACE2 receptor and inhibits the interaction of SARS-CoV-2 with this receptor. SARS-CoV-2, severe acute respiratory syndrome coronavirus 2; ACE2, angiotensin-converting enzyme 2; ROS, reactive oxygen species; TLR4, toll-like receptor 4; TRIF, TLR-like receptor-associated interferon factor; MyD88, myeloid differentiation factor 88; TRAF6, tumor receptor-associated factor 6; IKK, ikappaB kinase; NEMO, ikappaB kinase gamma; IRF, interferon regulatory factor; NF-κB, nuclear factor kappa B.

Pondering over the nutritional condition–immune response in patients with COVID-19, nutraceutical approaches that can ameliorate the immune system to protect or decrease the risk of serious progression and prognosis of coronavirus infection become pertinent (Figure 1). This review aims to give a brief insight and summarize the current literature regarding the utility of melatonin, zinc, and vitamin C as possible approaches for patients with COVID-19.

## Methods

### Search Question

The main question of the present review is: What are the completed randomized controlled trials (RCTs) and observational research, published in the literature, that studied melatonin, zinc, or vitamin C supplementation in the treatment of patients with COVID-19?

### Design and Identification of Relevant Studies

Due to the lack of concluded RCTs that assessed nutritional/nutraceutical treatments with melatonin, zinc, or vitamin C during the COVID-19 pandemic, this research is a narrative review with qualitative data in design but followed general guidelines used to establish sources from the literature. Thus, studies reporting RCTs or observational research were searched by PubMed database and the search plan explored Medical Subject Headings (MeSH) terms and free text search. The search terms and synonyms were divided into three main categories and lastly blended into one search sequence. Supplementary Table 1 shows the logical structure of the current search strategy with all the Boolean operators and descriptors used in the PubMed database. The reference lists of pertinent papers were also inspected to recognize additional suitable trials on the search criteria.

### Eligibility

Inclusion criteria were peer-reviewed RCTs and observational studies that evaluated patients with COVID-19 who underwent any treatment involving melatonin, zinc, or vitamin C; written in any language. Letters, editorials, and studies with a survey, questionnaire, case reports, or protocol without results were excluded. RCTs that used only a combination of supplements (e.g., ivermectin plus zinc) without the use of a specific treatment group involving melatonin, zinc, or vitamin C were also excluded. Moreover, research that studied patients with another type of coronavirus (e.g., MERS) as well as parenteral nutrition or trials that used melatonin, zinc, or vitamin C as a comparator control group, instead of using a placebo, were also excluded to avoid data confounding. The literature search was conducted from inception to October 04 (2021), with results being exported on the same day. Two independent reviewers read the titles and abstracts of the studies, and mutual conclusions were summarized. In cases of discrepancy, a third reviewer decided on the inclusion of the study. The restricted review was developed using RayyanQatar Computing Research Institute QCRI (http://rayyan.qcri.org) to select the included studies.

## Results and Discussion

### Characterization of Included Studies

Among 108 coronavirus-associated melatonin articles extracted and assessed for eligibility, three studies with a melatonin approach were included (33–35). Moreover, among the 399 coronavirus-associated zinc studies extracted and evaluated for eligibility, three studies entered the inclusion criteria (36–38), whereas among 344 coronavirus-associated vitamin C (or ascorbic acid) papers initially extracted and analyzed for eligibility, a total of 10 papers were also included as RCTs or observational research (37, 39–47).

Current observational research and RCTs on COVID-19 and nutraceutical approaches regarding melatonin, zinc, and vitamin C are summarized in Table 1 (melatonin), Table 2 (zinc), and Table 3 (vitamin C). Among the studies included, all reports included a control group that received placebo or standard therapy. Twelve studies were developed with patients with COVID-19 admitted to the hospital, two studies with ICU patients, and one study with ambulatory patients (Tables 1–3).

**Table 1:** Summary of findings from studies that included melatonin supplementation in the COVID-19 treatment.

**References**	**Study design, setting, gender, age**	**Therapy, regimen**	**N, severity of COVID-19, and comorbidities**	**Outcomes**
Mousavi et al. (33)	RCT; Hospital; Female (55.2%); 52.9 ± 1.8 years.	•Group 1 (*N* = 48): a single nightly 3 mg oral melatonin tablet for 7 days or until death. •Group 2 (*N* = 48): no supplementation (ST).	*N* = 96; Severity of COVID-19: NR; Comorbidity: DM (28.1%), asthma (5.2%) renal failure (10.4%), cardiovascular disease (15.6%), hypertension (30.2%), thalassemia (3.1%), thyroid disorders (8.3%), COPD (3.1%).	•LSEQ: ↑ melatonin group compared to CG. •WBC count, lymphocyte count, CRP: no difference on day 7. •Blood oxygen saturation: improved in the melatonin group. •On day 10: one patient from the melatonin group died vs. 3 patients in the CG.
Alizadeh et al. (34)	RCT; Hospital; Male (55.7%); 36.0 ± 1.5 years.	•Group 1 (*N* = 14): two tablets (6 mg) per day of melatonin for 2 weeks. •Group 2 (*N* = 17): no supplementation (ST).	N=31; Mild to moderate COVID-19; Comorbidity: Patients with comorbidities were excluded from the study.	•CRP1, CRP2: no difference between the groups. •% of the recovery in the melatonin group: higher in males. •Percentage of normal CRP: higher in females in the melatonin group but lower in the CG.
Farnoosh et al. (35)	RCT; Hospital; Male (59.1%); 51.8 ± 0.2 years.	•Group 1 (*N* = 24): 3 mg of melatonin three times daily for 14 days. •Group 2 (*N* = 20): no supplementation (ST).	*N* = 44; Mild to moderate COVID-19; Comorbidity: hypertension (25%), DM (23%), rheumatic disease (9%), cardiovascular disease (7%).	•Improvement of respiratory symptoms (coughs, dyspnea) and fatigue in the melatonin group compared to CG. •Improvement in the level of CRP, time of hospital discharge, and pulmonary involvement in the melatonin group compared to CG. •Melatonin group showed a return baseline health sooner compared to CG.

*COVID-19, coronavirus disease 2019; CG, control group; NR, not reported; DM, diabetes mellitus; CRP, C-reactive protein; mg, milligram; ST, standard therapy; LSEQ, Leeds sleep evaluation questionnaire; WBC, white blood cell; COPD, chronic obstructive pulmonary disease; ↑, increase*.

**Table 2:** Summary of findings from studies that included zinc supplementation in the COVID-19 treatment.

**References**	**Study design, setting, gender, age**	**Therapy, regimen**	**N, severity of COVID-19, and comorbidities**	**Outcomes**
Patel et al. (36)	RCT; Hospital; Male (64.4%); 61.8 ± 0.1 years.	•Group 1 (*N* = 15): 0.5 mg/kg/day of intravenous zinc chloride for a maximum of 7 days. •Group 2 (*N* = 18): saline placebo.	*N* = 33; Severity of COVID-19: NR. Comorbidity: hypertension (48.3%), DM (18.3%), cardiovascular (21.7%) and respiratory (11.1%) disease, cirrhosis (16.7%), hepatic failure (5.6%).	•↑ [serum zinc levels] in the intervention group, above the deficiency cutoff [10.7 μmol/l] 6 days after the supplementation, while the placebo group stayed below the cutoff.
Thomas et al. (37)	RCT; Ambulatory; Female (61.7%); 45.2± 14.6 years.	•Group 1 (*N* = 48): 8000 mg of VC (10 days). •Group 2 (*N* = 58): 50 mg of zinc gluconate (10 days). •Group 3 (*N* = 58): a combination of both therapies in groups 1 and 2 (10 days). •Group 4 (*N* = 50): no supplementation (ST).	*N* = 214; Moderate and Severe COVID-19; Comorbidity: DM (13.6%), hypertension (32.7%), dyslipidemia (26.2%), asthma (15.4%), anxiety (18.2%), depression (15.4%).	•Days required to reach 50% reduction in symptoms: No difference among the groups. •No distinction in the number of days to reach no presence of cough, fever, shortness of breath, or fatigue among the groups.
Abdelmaksoud et al. (38)	RCT; Hospital; Male (58.2%); 52.0 ± 12.2years.	•Group 1 (*N* = 49): 220 mg zinc sulfate equivocal to 50 mg elemental twice daily until complete recovery of COVID-19. •Group 2 (*N* = 56): no supplementation (ST).	*N* = 134; Mild, common, severe, and extremely severe COVID-19; Comorbidity: DM (16.1%), hypertension (11.7%), ischemic heart disease (6.1%).	•Duration of smell recovery: ↓ zinc group compared to CG. •Total recovery duration of COVID-19: no difference.

*COVID-19, coronavirus disease 2019; CG, control group; NR, not reported; mg: DM, diabetes mellitus; mg, milligram; kg, kilogram; μmol/l, micromole per liter; VC, vitamin C; ST, standard therapy; ↑, increase; ↓, decrease*.

**Table 3:** Summary of findings from studies that included vitamin C supplementation in the COVID-19 treatment.

**References**	**Study design, setting, gender, age**	**Therapy, regimen**	**N, severity of COVID-19, and comorbidities**	**outcomes**
Sulaiman et al. (42)	Cohort; ICU; Male (72%); 60.6 ± 14.8 years.	•Group 1 (*N* = 158): 1000 mg of VC enterally once daily with a median duration of administration of 11 days. •Group 2 (*N* = 581): no supplementation (ST).	*N* = 296; Severe COVID-19; Comorbidity: DM (59%), hypertension (56%), dyslipidemia (29%).	•There was no association between the administration of VC and in-hospital mortality or the 30-day mortality. •Thrombosis/infraction rate: ↓ in the VC group compared to CG.
Xia et al. (44)	RCT; Hospital; Female (55%); 67 ± 1years.	•Group 1 (*N* = 85): 100 mg/kg diluted 50 mL of saline solution on day 1 plus 100 mg/kg of body weight VC diluted in 50mL of saline solution for next 5 days. •Group 2 (*N* = 151): no supplementation (ST).	*N* = 236; severe COVID-19; Comorbidity: hypertension (15.0%), coronary heart disease (11.8%), DM (15.9%).	•CRP, IL-6, and tumor necrosis factor decreased in both groups, but the percentage of reduction in the VC group was better compared to CG. •VC group was independently associated with the percentage of reduction in levels of inflammatory markers.
Suna et al. (45)	Retrospective; Hospital; Male (63.1%); 62.2 ± 0.4 years.	•Group 1 (*N* = 153): 2 g/day intravenous VC. •Group 2 (*N* = 170): no supplementation (ST).	*N* = 323; Severe COVID-19; Comorbidity: hypertension (42%), DM (29.8), CAD (16.7%), heart failure (5.2%), COPD (14.15%), asthma (6.8), malignancy (9%), renal failure (3.35%), interstitial lung (0.65%) and rheumatological (3.15%) disease.	•CRP, D-dimer, and ferritin in the VC group between baseline and post-treatment: No difference between the groups. •Length of hospital stay, re-admission rate, admission to intensive care, need for advanced oxygen support, need for advanced medical treatment, and mortality: No difference between the groups. •Adverse effects: None reported.
Xia et al. (47)	Cohort; Hospital; Male (46%); 69.5 ± 1.5years.	•Group 1 (*N* = 51): 100 mg/kg of intravenous VC (1 day) followed by 100 mg/kg (5 days) during hospitalization. •Group 2 (*N* = 62): no supplementation (ST).	*N* = 113; Severe and critically COVID-19; Comorbidity: hypertension (45.3%), coronary heart disease (21.1%), DM (25.6%).	•VC group correlated (odds ratio 2.420, 95% CI 1.022–5.729) with the improvement of cardiac injury independent of mechanical ventilation and renal replacement therapy. •VC group improved myocardial damage among patients with SARS-CoV-2 infection in severe and critically ill conditions. •CRP, IL-6, IL-8, and tumor necrosis factor showed reduction at day 21 during hospitalization in the VC group compared to CG.
Kumari et al. (40)	RCT; Hospital; Male (56.9%); 52.5 ± 11.5 years.	•Group 1 (*N* = 75): 50 mg/kg/day of intravenous VC. •Group 2 (*N* = 75): no supplementation (ST).	N=150; severe COVID-19; Comorbidity: NR.	•VC group became symptom-free earlier than CG. •↓days hospitalized in the VC group (vs CG). •Need for mechanical ventilation and mortality: No difference.
JamaliMoghadam Siahkali et al. (41)	RCT; Hospital; Male (50%); 59.3 ± 17.1 years.	•Group 1 (*N* = 30): 1500 mg of intravenous VC for 5 days. •Group 2 (*N* = 30): CG (lopinavir/ritonavir and HCQ).	*N* = 60; Severe COVID-19; Comorbidity: hypertension (41.6%), DM (38.3), ischemic heart disease (18.3%), thyroid disease (8.3%), COPD (10%).	•↓mean body temperature and ↑SpO2 in the VC group on the 3rd day of hospitalization. •The length of hospitalization in the VC group was longer than the CG. •ICU stay, mortality rate, and intubation: No difference. •Fever and myalgia were less frequent in the VC group.
Zhang et al. (39)	RCT; ICU; Male (66.1%); 66.7 ± 12.7years.	•Group 1 (*N* = 27): 24 g of intravenous VC per day for 7 days. •Group 2 (*N* = 29): placebo (bacteriostatic water infusion).	*N* = 56; Severe COVID-19 Comorbidity: DM (30.4%), hypertension (44.6%), coronary heart disease (21.4%), chronic lung disease (5.4%), chronic renal failure (1.8%), malignant tumor (5.4%), nervous system diseases (20.4%).	•No difference in days of absence (in 28 days) of mechanical ventilation between both groups. •VC group exhibited a trend (p=0.06) of reduction in 28-day mortality in more severe patients (SOFA score ≥3).•SOFA score: ↓ in the VC group, ↑ in the placebo group. •IL-6: ↓ in the VC group and ↑ in the placebo group. •Infectious indicators: No difference.
Thomas et al. (37)	RCT; Ambulatory; Female (61.7%); 45.2± 14.6years.	•Group 1 (*N* = 48): 8000 mg of VC (10 days). •Group 2 (*N* = 58): 50 mg of zinc gluconate (10 days). •Group 3 (*N* = 58): a combination of both therapies in groups 1 and 2 (10 days). •Group 4 (*N* = 50): no supplementation (ST).	*N* = 214; Moderate and Severe COVID-19; Comorbidity: DM (13.6%), hypertension (32.7%), dyslipidemia (26.2%), asthma (15.4%), anxiety (18.2%) depression (15.4%).	•Days required to reach 50% reduction in symptoms: No difference among the groups. •No distinction in the number of days to reach no presence of cough, fever, shortness of breath, or fatigue among the groups.
Zhao et al. (43)	Retrospective BACMCS; Hospital; Male (61.8%); 36 years.	•Group 1 (*N* = 55): 100 mg/kg/day for 7 days from admission for one month. •Group 2 (*N* = 55): no supplementation (ST).	*N* = 110; Moderate COVID-19; Comorbidity: hypertension (6.4%), DM (6.4%).	•VC group: less incidence of the final diagnosis of severe or critical COVID-19. •On Day 7, there were fewer patients with SIRS in the VC group compared to CG. However, the duration of SIRS was significantly shorter in the VC group compared to CG. •On Day 7, CRP levels: ↓ in VC group compared to CG. •VC group: improved effect on the CD4+ T lymphocyte deficiency on admission. •There were no obvious effects of the VC therapy of CD4+ T cell counts, CD8+ T cell counts, and lymphocytes counts on days 3 and 7 for the entire study population. •D-dimer levels: ↓VC group compared to CG. •APTT in the VC group (seconds) was significantly shorter than in the CG.
Gao et al. (46)	Cohort; Hospital; Male (46.1%); 61 years.	•Group 1 (*N* = 46): 6 g of intravenous VC (1° day), plus 6 g of VC per day (4 days). •Group 2 (*N* = 30): no supplementation (ST).	*N* = 76; Moderate and severe COVID-19; Comorbidity: DM (19.7%), hypertension (28.9%), coronary heart disease (6.6%), underlying lung disease (7.9%), chronic liver disease (5.3%), chronic kidney disease (2.6%).	•Risk of mortality: ↓VC group compared to CG. •In the VC group, clinical improvement was better for patients younger than 60 years old. Moreover, the VC treatment was better for patients who received low-flow oxygen, and those with CRP <1 mg/L than their counterparts. •CRP, procalcitonin, and IL-8 levels: ↓ in the VC group compared to CG. •Thrombocytopenia and increased total bilirubin events were common in both groups. However, the incidence was lower in the VC group compared to CG. •Six patients showed serious adverse events (respiratory failure or ARDS, shock, and sepsis): one in the VC group and five in the CG. Moreover, respiratory failure or ARDS were more common in the CG.

*COVID-19, coronavirus disease 2019; CG, control group; NR, not reported; CAD, Coronary artery disease; DM, diabetes mellitus; mg: milligram; kg, kilogram; mL, milliliter; g, gram; HCQ, hydroxychloroquine; VC, vitamin C; SpO_2_, peripheral capillary oxygen saturation; IL, interleukin; ICU, intensive care unit; SOFA, sequential organ failure assessment; ST, standard therapy; SIRS, systemic inflammatory response syndrome; CRP, C-reactive protein; APTT, activated partial thromboplastin time; ARDS, acute respiratory distress syndrome; SARS-CoV-2, severe acute respiratory syndrome coronavirus 2; LSEQ: COPD, chronic obstructive pulmonary disease; Leeds sleep evaluation questionnaire; WBC, white blood cell; CI, confidence interval; ↑, increase; ↓, decrease*.

### Melatonin

Besides the indirect antiviral responses due to melatonin immunomodulatory, antioxidant, antiinflammatory, and immune-enhancing characteristics (48), recent multidrug repurposing research on 26,779 participants affected by COVID-19 showed that increased melatonin levels were related to a 28% (general population) and 52% (African Americans) decreased likelihood of a SARS-CoV-2 infection (49).

Randomized controlled trials are the gold standard for clinical studies, and they are crucial for establishing whether there is a real clinical advantage in using therapy to manage COVID-19. Clinical trials that preceded the COVID-19 pandemic found adequate safety outcomes when this endogenous indolamine was orally supplemented at different concentrations (3, 6, and 10 mg) in patients from intensive care units (ICU) (50, 51). Currently, eight clinical studies with melatonin treatment (or melatonin agonist therapy) in patients with COVID-19 are reported at clinicaltrials.gov. In highlight, three of them have already initiated enrolling subjects, and two trials have the recruitment status of “complete” (NCT04570254, NCT04568863) (Supplementary Table 2).

In the context of the COVID-19 pandemic, Farnoosh et al. developed a small (*N* = 44) RCT to study a group of patients with COVID-19 treated additionally with melatonin (3 mg for 2 weeks) and found a more rapid hospital discharge and return to baseline health in the melatonin group, as well as relevant improvement in the pulmonary involvement and clinical symptoms such as cough, dyspnea, and fatigue, and also the level of CRP (35). Corroborating the findings, a pilot randomized study (*N* = 31) in patients with COVID-19 also showed that melatonin intervention (6 mg for 2 weeks) increased the percentage of recovery (based on symptoms) when compared with patients in the control group (34). Moreover, Mousavi et al. assessed the effectiveness of adding melatonin treatment (3 mg plus standard care for 7 days or until death) on sleep quality and outcomes of patients with COVID-19 (*N* = 96). They found that the mean of the Leeds Sleep Evaluation Questionnaire scores was higher in the melatonin group; however, there was no difference in laboratory data (lymphocyte count, white blood cell count, CRP levels), except for blood oxygen saturation, which has improved in the melatonin group (33).

### Vitamin C

Before the current pandemic, clinical outcomes have emphasized a relevant function for vitamin C among individuals in ICU with pneumonia, sepsis, ARDS, and multiorgan failure (52). For instance, a meta-analysis of 12 RCTs showed that ascorbic acid low ICU permanence on average by 8% in sepsis condition or cardiac surgery patients involving different clinical conditions (e.g., atrial fibrillation and coronary artery bypass grafting patients) (53). The results of vitamin C on distinct groups of respiratory viruses appear to be non-specific (54); therefore, it seems reasonable that this nutritional therapy may also have outcomes on the new coronavirus.

Vitamin C treatment appears to have a partial clinical improvement effect in patients with COVID-19. For instance, the pilot trial developed by Zhang et al. found that HDIVC [24 g; *N* = 27] failed to ameliorate the invasive mechanical ventilation-free days in 28 days when compared to the control group [*N* = 29]. On the other hand, the authors showed that HDIVC reduced IL-6 levels, and the treatment might also show a possible signal of benefit in oxygenation by the improvement of partial pressure of oxygen (PaO_2_/FiO_2_) for severely ill patients with COVID-19 (39). Similarly, Kumari et al. showed that the treatment with intravenous vitamin C in patients with COVID-19 also failed to reduce mortality and the necessity for mechanical ventilation between the therapy [50 mg/kg/day; *N* = 75] and the control group [*N* = 75]; however, they found that vitamin C therapy allowed patients with COVID-19 a shorter hospital stay, and these patients became symptom-free earlier compared to patients using standard treatment only (40).

Thomas et al. did not find a decrease in the duration of symptoms in patients with SARS-CoV-2 infection supplemented with vitamin C [8,000 mg for 10 days; *N* = 48] (37). On the other hand, Jamali Moghadam Siahkali et al. showed amelioration in body temperature and peripheral oxygen saturation in serious patients with COVID-19 with HDIVC therapy [6g/day; *N* = 30]. However, the authors did not find better outcomes in the vitamin C group in addition to the main treatment regimen at discharge (41).

Although a few observational research and preliminary clinical studies on vitamin C in patients with COVID-19 corroborate their findings by noting a reduction in hyper inflammation (43, 44, 47), improvement in the oxygen support status (39, 41, 46, 47), lower incidence of thrombosis (42), and also the reduced disease aggravation in the early stage of COVID-19 pneumonia (43), the mortality rate is still controversial among the studies. Some authors found a reduction in mortality (46) whereas other studies with vitamin C did not observe a difference in mortality when compared to patients in the control group (40, 42, 45). Controversial research with vitamin C also demonstrated failure to ameliorate the mechanical ventilation of patients with COVID-19 (39, 40) and did not find a substantial decrease in the duration of symptoms (37). Therefore, further studies are required to confirm these findings since limited evidence based on small samples precludes definitive conclusions.

Currently, more than 60 ongoing trials are studying SARS-CoV-2 and oral and/or intravenous ascorbic acid treatment, twenty-four of which have started the recruitment of participants, and 15 of which have already been finished (Supplementary Table 2).

### Zinc

Zinc treatment investigation is rational in COVID-19 since reduced zinc concentrations seem frequent in patients with COVID-19 (55, 56) and due to the protective response of zinc on viral replication. In this sense, zinc seems to restrain coronavirus RNA polymerase response *in vitro* (20), which could grant this metal a function in avoiding virus entrance into cells and decreasing the virulence of SARS-CoV (57). Moreover, zinc toxicity hardly happens in sporadic cases in contrast to numerous other metal ions with close chemical features (58).

Interestingly, patients with COVID-19 with decreased zinc concentrations demonstrated higher mortality on hospital admission (55). Thus, it has been hypothesized that the temporary zinc deficit that takes place in COVID-19 infection could produce a hyperinflammatory state. On the other hand, the antiinflammatory response of zinc has been evidenced by the restriction of IkappaB kinase response, modulation of T-cell function, and NF-κB signaling with a simultaneous decrease in the concentrations of proinflammatory biomarkers (59). Moreover, zinc therapy is suggested to decrease inflammatory cytokines (IL-1 and IL-6), an event that could improve the protective type-I IFN response in COVID-19 (7).

Patel et al. performed a pilot trial (*N* = 33) in hospitalized COVID-19 patients and found high serum zinc levels in the intervention group supplemented with zinc [0.5 mg/kg/day] (above the deficiency cutoff of 10.7 μmol/l) 6 days after the treatment, while the placebo group stayed below the cutoff (36). However, Abdelmaksoud et al. showed that the zinc status of patients with COVID-19 did not exhibit a relevant role in the disease severity or in the development of hyposmia and/or anosmia. The same authors also found that the median duration of recovery of olfactory and/or gustatory role was shorter among patients with COVID-19 who had zinc therapy (*N* = 49; 220 mg zinc sulfate equivocal to 50 mg elemental zinc two times daily, until complete recovery of COVID-19) than those who did not receive zinc (*N* = 56) (38).

Thomas et al. studied patients treated with high-dose zinc gluconate [50 mg; *N* = 58] for 10 days in ambulatory patients with SARS-CoV-2 infection with the primary endpoint being the number of days needed to reach a 50% decrease in symptoms, such as the severity of fatigue, cough, fever, and shortness of breath. They found no decreases in the duration of symptoms in patients supplemented with high-dose zinc when compared with the standard of care (*N* = 50). In fact, the authors had to interrupt the trial due to the low conditional power for benefit among the participants (37).

To date, contrasting and scarce clinical outcomes on zinc treatment efficacy on COVID-19 are accessible, especially in the outpatient setting. It is also pertinent to note that numerous clinical protocols planned to utilize zinc in association with azithromycin or hydroxychloroquine (e.g., NCT04370782, NCT04377646), and it is unknown how the absence of proof encouraging the utilization of hydroxychloroquine will reflect in research with zinc treatment.

Presently, more than 50 trials (more than 15 identified with “in recruitment” status and “completed” status) are reported with the purpose to use zinc supplementation in a therapeutic or preventative way, and the outcomes of these trials will be significant to authenticate the efficiency of zinc as a secondary approach in SARS-CoV-2 (Supplementary Table 2).

### Final Considerations

Besides the fact that nutritional supplementation is usually cost-effective, research with melatonin, zinc, and vitamin C are reported with antiinflammatory and antioxidative attributes, and the deficiency of these nutraceutical supports represents a higher risk for severe progression of COVID-19. Our data showed that, to date, most RCTs with the use of melatonin, zinc, or vitamin C in patients with COVID-19 are still in the development stage and, even though some trials already have the status of “completed,” as demonstrated in Supplementary Table 2, there is still no forecast for the publication of these studies.

Considering only the data with a statistical difference, we could hypothesize that zinc therapy has the potential to increase the serum zinc levels (above the deficiency cutoff) (36) and could promote shorter duration in the recovery of gustatory and/or olfactory function in patients with COVID-19 (38). Furthermore, the hypothesis involving the treatment with melatonin in patients with COVID-19 may suggest improved blood oxygen saturation and better sleep quality (33), as well as faster hospital discharge and improvement in the clinical symptoms (34, 35). However, the studies mentioned have inconclusive data with a relatively small sample size and early termination of the trial.

Among the research that used vitamin C therapy, there appears to be an agreement in observing a reduction in hyperinflammation (39, 43, 44, 47) and improvement in the oxygen support status of patients with COVID-19 (39, 41, 46, 47). However, there are still conflicting data regarding mortality rate, mechanical ventilation, and duration of the symptoms of patients with COVID-19. One important limitation among the studies was the lack of reports around the baseline vitamin C status of the patients with COVID-19. Since there is a great change in dietary vitamin C intake and thereafter in baseline plasma concentrations, if baseline concentrations are elevated, then the therapy with vitamin C could be less probable to promote an effect than when baseline concentrations are low.

Another important limitation of the studies in this review is related to the gender imbalance among the participants. Since the inflammatory response is influenced by sex (60) and there were a greater number of studies with male participants, caution should be exercised when extrapolating the findings to both genders. In addition, due to the lack of trials presenting zinc supplementation without combination with hydroxychloroquine, this review included only three studies using zinc therapy in patients with COVID-19. It is also worth mentioning that one of these studies had to discontinue the trial due to the low conditional power for benefit related to COVID-19 symptoms (37).

The lack of data on the monitoring of melatonin, zinc, and vitamin C therapy made it unclear whether these nutraceutical approaches have a direct antiviral response against SARS-CoV-2. Besides, a direct comparative investigation of the nutraceutical approaches comparing intravenous vs. oral supplementation can benefit current literature. Therefore, caution should be taken when extrapolating the outcomes on a larger scale, and additional data from the ongoing trials are needed to acquire more reports on the application of these molecules as a prevention and/or therapy in the present pandemic.

## Author Contributions

LB developed the idea and wrote the manuscript. BD and MG-F collected and prepared the study data. EH reviewed and edited the manuscript. All authors have read and agreed to the published version of the manuscript.

## Funding

This work was supported by the CAPES (Coordenação de Aperfeiçoamento de Pessoal de Nível Superior) [grant numbers 88882.314890/2013-01 and 88882.365204/2019-01, Finance Code 001]; and CNPq (Conselho Nacional de Desenvolvimento Científico e Tecnológico) [grant number 308700/2017-1].

## Conflict of Interest

The authors declare that the research was conducted in the absence of any commercial or financial relationships that could be construed as a potential conflict of interest.

## Publisher's Note

All claims expressed in this article are solely those of the authors and do not necessarily represent those of their affiliated organizations, or those of the publisher, the editors and the reviewers. Any product that may be evaluated in this article, or claim that may be made by its manufacturer, is not guaranteed or endorsed by the publisher.
