# Supplementary material for: Melatonin, Zinc, and Vitamin C: Potential Adjuvant Treatment for COVID-19 Patients
Source: Front Nutr. 2022 Jan 26;8:821824. doi: 10.3389/fnut.2021.821824 (PMC8826215; doi:10.3389/fnut.2021.821824)
Supplement: Supplementary file 1 [file Table_1.pdf]

SUPPLEMENTARY MATERIAL

Table S1. Electronic search strategy on PubMed database.

**Electronic search strategy**
1. COVID-19 OR coronavirus OR SARS-CoV-2
2. intervention OR trial OR randomized controlled trial OR RCT or observational
3. melatonin OR N-acetyl-5-methoxytryptamine OR zinc OR vitamin C OR ascorbic acid
4. (#1) AND (#2) AND (#3) AND

Note: The literature search was conducted from inception to October 04 (2021).
