# Supplementary material for: Melatonin, Zinc, and Vitamin C: Potential Adjuvant Treatment for COVID-19 Patients
Source: Front Nutr. 2022 Jan 26;8:821824. doi: 10.3389/fnut.2021.821824 (PMC8826215; doi:10.3389/fnut.2021.821824)
Supplement: Supplementary file 2 [file Table_2.pdf]

SUPPLEMENTARY MATERIAL

Table S2. Studies involving randomized controlled trials with nutritional/nutraceutical approaches for the therapy in COVID-19 patients.

**Melatonin**										
**Identifier**	**Intervention/treatment**	**Primary Outcome Measures**	**Last Update Posted**	**n**	**Prim. Purp.**	**Start Date**	**Estim. Compl. Date**	**Ages**	**Status**
NCT04474483	Melatonin; Placebo (Methylcellulose)	Cumulative Incidence of Treatment-Emergent Adverse Events	Nov 2020	30	T	Nov 2020	Mar 2021	≥18	Recruiting
NCT04784754	Melatonin; Placebo	Cumulative Incidence of Treatment-Emergent Adverse Events	Mar 2021	50	T	Mar 2021	Dec 2021	≥18	Not yet recruiting
NCT04531748	Toremifene; Melatonin; Placebo	Peak increase in COVID-19 Sign and Symptom score	May 2021	0	T	Aug 2020	Sep 2022	>18	Withdrawn (Funding)
NCT04568863	Melatonin intravenous; Placebo intravenous	Mortality	Mar 2021	18	T	Jun 2020	Nov 2020	≥18	Completed
NCT04530539	Vitamin C; melatonin; Placebo; Symptom Survey	Symptom Severity	Feb 2021	150	SC	Oct 2020	Dec 2021	>50	Recruiting
NCT04353128	Melatonin; Placebo	SARS-CoV2 infection rate	Jun 2020	450	P	Ap 2020	Dec 2020	18-65	Recruiting
NCT04470297	Ramelteon (melatonin agonist)	Time to resolution of symptoms	Jul 2020	100	T	Sep 2020	Mar 2021	18	Not yet recruiting
NCT04570254	Vitamin C; Vitamin E; Melatonin; Drug: N-acetyl cysteine; Pentoxifylline	Death from any cause	May 2021	110	T	Aug 2020	Dec 2020	All ages	Completed

Vitamin C									
**Identifier**	**Intervention/treatment**	**Primary Outcome Measures**	**Last Update Posted**	**n**	**Prim. Purp.**	**Start Date**	**Estim. Compl. Date**	**Ages**	**Status**
NCT04664010	Alpha-interferon alpha, abidol, ribavirin, Buzhong Yiqi plus and minus formula, Huhuang Detoxicity Paste, Baimu Qingre Jiedu Paste, fumigation/inhalation of vitamin C; Alpha-interferon, abidol, ribavirin, Buzhong Yiqi plus and minus formula, Huhuang Detoxicity Paste, Baimu Qingre Jiedu Paste and 5% glucose; Alpha-interferon, abidol, ribavirin, Buzhong Yiqi plus and minus formula, Huhuang Detoxicity Paste, Baimu Qingre Jiedu Paste and high-dose vitamin C treatment	Recovery time	Dec 2020	60	T	Feb 2020	Jan 2021	18-70	Active, not recruiting
NCT04363216	Ascorbic Acid	Clinical Improvement	May 2020	66	T	May 2020	May 20221	18	Not yet recruiting
NCT04347889	Hydroxychloroquine; Vitamin C	COVID-19 Seroconversion rate	Nov 2020	0	P	Apr 2020	Dec 2020	18	Withdrawn (Evidence that HCQ ineffective, loss of HCW interest)
NCT04323514	Vitamin C	In-hospital mortality	Mar 2020	500	T	Mar 2020	Mar 2021	All Ages	Recruiting
NCT04710329	Ascorbic acid	Short term mortality; Length of Intensive Care Unit Stay	Feb 2021	78	R	Jan 2021	Feb 2021	18-99	Completed
NCT04530539	Vitamin C; melatonin; Placebo; Symptom Survey	Symptom Severity	Feb 2021	150	SC	Oct 2020	Dec 2021	50	Recruiting
NCT04558424	Zinc gluconate and ascorbic acid	symptoms reduction time frame	Sep 2020	50	SC	Oct 2020	Sep 2021	18-70	Not yet recruiting
NCT04468139	Quercetin; bromelain; Zinc; Vitamin C	Days of stay at hospital after treatment and discharge; serum zinc before and after treatment	Jul 2020	60	T	Jun 2020	Jul 2020	18	Recruiting
NCT04357782	L-ascorbic acid	Incidence of adverse events; Incidence of serious adverse reactions; Incidence of adverse reactions;	Dec 2020	20	T	Apr 2020	Oct 2020	18-99	Completed
NCT04395768	Vitamin C; Hydroxychloroquine; Azithromycin; Zinc Citrate; Vitamin D3; Vitamin B12	Symptoms; Length of hospital stay; invasive mechanical ventilation or mortality	Sep 2020	200	T	Sep 2020	Dec 2021	18	Recruiting
NCT04401150	Vitamin C; Control	Death or persistent organ dysfunction	Mar 2021	800	T	Aug 2020	Jan 2022	18	Recruiting
NCT04342728	Ascorbic Acid; Zinc Gluconate; Ascorbic Acid and Zinc Gluconate; Standard of Care	Symptom Reduction	Feb 2021	214	SC	Apr 2020	Feb 2021	18	Completed

NCT04828538	Vitamin D; Omega DHA / EPA; Vitamin C, Vitamin B complex and Zinc Acetate	Efficacy for COVID infection prevention; Efficacy for treatment of severe COVID disease	Apr 2021	3600	O	Mar 2021	Jul 2021	18	Recruiting
NCT04344184	L-ascorbic acid; Placebo	Change in COVID disease status	Jun 2021	60	T	Dec 2022	Mar 2022	18	Recruiting
NCT04483960	Nafamostat Mesilate; Convalescent plasma; Enoxaparin; Dalteparin; Tinzaparin; Aspirin	Death from any cause or requirement of new intensive respiratory support (invasive or non-invasive ventilation) or vasopressor/inotropic support.	Jun 2021	2400	T	Jul	Dec 2022	18	Recruiting
NCT04712357	Vitamin C, Oral Tablet; Tenofovir disoproxyl fumarate; Tenofovir disoproxyl fumarate plus emtricitabine.	The time to recovery, defined at day 7 days follow up after enrollment, on which a patient met the criteria for category 1, 2, or 3 on the eight-category ordinal scale	Jan 2021	219	T	Nov 2020	Jan 2023	18	Recruiting
NCT04780061	Vitamin D; Vitamin C/Zinc; Vitamin K2/D; Microcrystalline Cellulose Capsule; Medium Chain Triglyceride Oil	Participant-reported overall health	Mar 2021	200	T	Apr 2021	Dec 2021	18	Not yet recruiting
NCT04370288	MCN (Methylene blue, vitamin C, N-acetyl cysteine)	Proportion of patients remaining free of need for mechanical ventilation in both groups	May 2020	20	T	Apr 2020	Sep 2020	18-90	Recruiting
NCT04335084	Hydroxychloroquine; Vitamin C; Vitamin D; Zinc	Prevention of COVID-19 symptoms as recorded in a daily diary; Safety as determined by presence or absence of Adverse Events and Serious Adverse Events	Feb 2021	600	P	Jun 2020	Dec 2021	18	Recruiting
NCT04584437	Infrared Energy and Dietary Supplement	Treatment and Prevention of COVID-19 Pandemic	May 2021	0	T	May 2021	Jul 2022	All Ages	Withdrawn (Funding)
NCT04682574	Vitamin C	Partial pressure of Oxygen in arterial blood to fraction of inspired Oxygen (P/F ratio)	Dec 2020	15	T	Nov 2020	Jan 2021	All Ages	Recruiting
NCT04334967	Hydroxychloroquine; Vitamin C	Total Hospitalization; Total Mechanical Ventilation	Sep 2020	13	T	Mar 2020	May 2022	45	Suspended (suspected unfavorable risk/benefit assessment)
NCT04328961	Hydroxychloroquine Sulfate; Ascorbic Acid	Polymerase chain reaction (PCR) confirmed SARS-CoV-2 infection; Polymerase chain reaction (PCR) confirmed SARS-CoV-2 infection	Oct 2020	829	P	Mar 2020	Oct 2020	18	Completed
NCT04830852	---	Incidence and prevalence of medical sequelae; Risk factors for medical sequelae.	Apr 2021	5000	P	May 2021	May 2027	>21	Not yet recruiting
NCT04264533	VC; Sterile Water for Injection	Ventilation-free days	Oct 2020	56	T	Feb 2020	Mar 2020	18	Terminated
NCT04279197	Fuzheng Huayu Tablet; Vitamin C tablets; Placebo; respiratory function rehabilitation training	The improvement proportion of pulmonary fibrosis	Sep 2020	160	T	Apr 2020	Dec 2021	18-70	Recruiting
NCT04446104	Hydroxychloroquine Sulfate; Ivermectin; Zinc; Povidone-Iodine; Vitamin C	Laboratory-confirmed COVID-19 in treatment arms	Oct 2020	4257	P	May 2020	Aug 2020	21-60	Completed
NCT04570254	Vitamin C; Vitamin E; Melatonin; N-acetyl cysteine; Pentoxifylline	Death from any cause; Percentage of patients who required orotracheal intubation; Assisted mechanical ventilation; Stay in an intensive care unit	May 2021	110	T	Aug 2020	Dec 2020	All Ages	Completed
NCT04631536	Atorvastatin + L-arginine + Folic acid + Nicorandil + Nebivolol; Placebo	Clinical Improvement	Mar 2021	80	T	Jan 2021	Jun 2021	18	Recruiting
NCT04334512	Hydroxychloroquine; Azithromycin; Vitamin C; Vitamin D; Zinc	The rate of recovery of mild or moderate COVID-19 in patients using Quintuple Therapy; Reduction or Progression of Symptomatic Days; Assess the safety of Quintuple Therapy; Assess the safety of Quintuple Therapy via pulse; Assess the	Apr 2021	600	T	Jun 2020	Sep 2021	18	Recruiting

		safety of Quintuple Therapy via oxygen saturation; Assess the safety of Quintuple Therapy via EKG; Assess Tolerability of Quintuple Therapy							
NCT04668469	Ivermectin; Hydroxychloroquine; personal protective Measures	Number of participants with improvement of clinical condition (symptoms and signs); Reduction of recovery time, hospital stay days and mortality rate	Dec 2020	600	T	Jun 2020	Oct 2020	18-80	Completed
NCT04354428	Ascorbic Acid; Hydroxychloroquine Sulfate; Azithromycin; Folic Acid; Lopinavir/ Ritonavir [Kaletra]	Lower respiratory tract infection rates; Incidence of hospitalization or mortality; Change in upper respiratory viral shedding; COVID-19 symptom resolution rates [Lopinavir-ritonavir arm only]	Nov 2020	300	T	Apr 2020	Jan 2021	18-80	Active, not recruiting
NCT04802382	combination of Artemisinin 12 mg, Curcumin 40 mg, Boswellia 30 mg, and Vitamin C 120 mg in spray administration	Clinical improvement in treatment groups	Mar 2021	252	T	Apr 2021	Dec 2021	18	Not yet recruiting
NCT04482686	Ivermectin; Doxycycline Hcl; Zinc; Vitamin D3; Vitamin C	Time to Non-Infectivity by RT-PCR; Time to Symptom progression in days as measured by NEWS scoring system (National Early Warning Score); Time to Symptom improvement as measured by NEWS scoring system (National Early Warning Score); Efficacy of Treatment as measured by Titer; Efficacy of Treatment as measured by RT-PCR.	May 2021	30	T	Dec 2020	Jul 2022	18-75	Recruiting
NCT04590274	Hydroxychloroquine; Vitamins and Minerals; Azithromycin	Percentage of individuals who develop COVID-19 symptoms	Oct 2020	5000	P	Nev 2020	Dec 2021	All Ages	Not yet recruiting
NCT04554979	Hydroxychloroquine	COVID-19 disease spectrum and duration; GIT manifestations among COVID-19 patients.	Sep 2020	199	R	Jun 2020	Jul 2020	18	Completed
NCT04877509	Selenium, Zinc and Copper, Vitamin A, D, E plasma concentrations during patient hospitalization	Selenium, Zinc and Copper, Vitamin A, D and E plasma concentrations of 229 over 50 year's patients hospitalized suffering from COVID-19.	May 2021	229	O	Mar 2020	May 2021	50	Completed
NCT04326725	Plaquenil	Protection against COVID-19	Jul 2020	80	P	Mar 2020	Sep 2020	20-90	Active, not recruiting
NCT04444271	Mesenchymal stem cells; Placebo	Overall survival	Jun 2020	20	T	May 2020	Sep 2020	10	Recruiting
NCT04382040	ArtemiC; Placebo	Time to clinical improvement, defined as a national Early Warning Score 2 (NEWS2) of </= 2 Maintained for 24 Hours in comparison to routine treatment; Percentage of participants with definite or probable drug related adverse events	Mar 2021	50	T	May 2020	Nov 2020	18	Completed
NCT04751669	Vitamin and trace elements; Placebo	Need for hospital admission	Apr 2021	300	T	Apr 2021	Dec 2021	18-80	Not yet recruiting
NCT04777981	CBDRA60 supplement; Placebo	Decreased hospitalization	Mar 2021	60	T	Apr 2021	May 2021	18	Not yet recruiting
NCT04558463	Favipiravir; Oseltamivir	Clinical radiologic changes; Percentage of RT-PCR test convertion	Sep 2020	100	T	Apr 2020	Oct 2020	18-75	Recruiting
NCT04551755	Ivermectin and Doxycycline; Placebo	Time to outcome measure of fever (<100.40F) and cough; Negative RT-PCR test on day 5 of treatment	Sep 2020	188	T	Sep 2020	Dec 2020	18	Not yet recruiting
NCT02735707	Fixed-duration Hydrocortisone; Shock-dependent hydrocortisone; Ceftriaxone; Moxifloxacin or Levofloxacin; Piperacillin-tazobactam; Ceftaroline; Amoxicillin-clavulanate; Macrolide; Macrolide administered; Oseltamivir	All-cause mortality<br>Days alive and not receiving organ support in ICU	Oct 2020	7100	T	Apr 2016	Dec 2023	18	Recruiting

NCT04690920	Tocilizumab	Ratio of partial pressure of oxygen in arterial blood to fraction of inhaled oxygen	Dec 2020	200	SC	Jul 2020	Dec 2020	All Ages	Completed
NCT04492501	Procedure: Therapeutic Plasma exchange; Biological: Convalescent Plasma; Drug: Tocilizumab; Drug: Remdesivir Biological: Mesenchymal stem cell therapy	Survival	Jul 2020	600	T	Apr 2020	Jul 2020	18-90	Completed
NCT04409483	Drug: Lopinavir-Ritonavir Drug Combination Combination Product: Standard Care	Hospitalization or death	Feb 2021	0	T	Jun 2020	Dec 2020	12	Withdrawn (Epidemic dynamics)
NCT04323228	Dietary Supplement: Oral supplement enriched in antioxidants Dietary Supplement: cellulose-containing placebo capsules	Score of Nutrition risk screening-2002 (NRS-2002); Serum ferritin; serum Interleukin-6 concentration; serum C-reactive protein concentration; serum Tumor necrosis factor-α concentration; serum monocyte chemoattractant protein 1.	Sep 2020	40	SC	Sep 2020	Dec 2020	18-65	Recruiting
NCT04360980	Drug: Colchicine Tablets	CRPxN/R ratio change; Clinical deterioration by the WHO definition; PCR Viral Load; CT severity involvement index	Dec 2020	80	T	Mar 2020	Nov 2021	18	Recruiting
NCT03680274	Drug: Vitamin C Other: Control	Number of deceased participants or with persistent organ dysfunction	Mar 2021	800	T	Nov 2018	Dec 2022	18	Recruiting
NCT04591600	Drug: Ivermectin and Doxycyline Drug: Standard of care	Mortality rate; Rate of progression disease	Oct 2020	140	T	Jul 2020	Oct 2020	16-86	Completed
NCT04666753	Dietary Supplement: ImmunoFormulation	Clinical symptoms duration.	Dec 2020	40	R	Jul 2020	Sep 2020	18	Completed
NCT04361422	Drug: Isotretinoin Only Product in Oral Dose Form	Clinical clearance	Sep 2020	300	T	Dec 2020	Dec 2021	18-40	Not yet recruiting
NCT04708340	Drug: Rejuveinix (RJX) Active Comparator; Drug: Placebo Comparator	Tolerability and Efficacy measured by progression of disease through an ordinal scale; Efficacy measured by time to resolution of respiratory failure	May 2021	237	T	Mar 2021	Feb 2023	18	Recruiting
NCT04853199	Drug: Quercetin; Drug: Placebo	The Efficacy measurement	Jun 2021	200	T	Jun 2021	Aug 2021	18	Not yet recruiting
NCT04551339	Dietary Supplement: PreserVision AREDS formulation soft gels or tablets; Dietary Supplement: Multivitamin with 11mg of zinc	COVID-19 illness requiring hospitalization	Mar 2021	4500	O	Sep 2020	Sep 2021	18-90	Active, not recruiting
NCT04507867	Dietary Supplement: Nutritional support system (NSS); Other: Control	Complications in patients with COVID-19 in stage III with comorbidities	Aug 2020	240	SC	Aug 2020	Dec 2020	35-75	Not yet recruiting
NCT04353180	Isotretinoin (13 cis retinoic acid); capsules+standard treatment; Isotretinoin (Aerosolized 13 cis retinoic acid) +standard treatment; Isotretinoin (13 cis retinoic acid); Aerosolized 13 cis retinoic acid; Standard treatment	Lung injury score	Apr 2021	1000 00	T	Apr 2021	Jul 2021	18-80	Not yet recruiting
NCT04851821	Drug: Quercetin	Number of patients	Jun 2021	80	T	Jan 2021	Jun 2021	18	Recruiting

Zinc									
**Identifier**	**Intervention/treatment**	**Primary Outcome Measures**	**Last Update Posted**	**n**	**Prim. Purp.**	**Start Date**	**Estim. Compl. Date**	**Ages**	**Status**
NCT04641195	Vitamin D3 (cholecalciferol); Zinc (zinc gluconate); Zinc (zinc gluconate) & Vitamin D (cholecalciferol); Placebo	Time to recovery	Ap 2021	700	T	Ap 2021	Mar 2022	>=18	Recruiting
NCT04558424	zinc gluconate and ascorbic acid	symptoms reduction time frame	Sep 2020	50	SC	Oct 2020	Sep 2021	18-70	Not yet recruiting
NCT04828538	Vitamin D; Omega DHA / EPA; Vitamin C, Vitamin B complex and Zinc Acetate	Efficacy for COVID infection prevention; Efficacy for treatment of severe COVID disease	Ap 2021	3600	O	Mar 2021	Jul 2021	≥18	Recruiting
NCT04370782	Hydroxychloroquine; Azithromycin; Zinc Sulfate; Doxycycline	Time to Resolution of Symptoms relative to baseline; Time to Resolution of Symptoms relative to baseline; Time to Resolution; Number of participants hospitalized and/or requiring repeat ER visits; Number of participants hospitalized and/or requiring repeat ER visits; ICU Length of Stay; Ventilator.	Dec 2020	18	T	Apr 2020	Sep 2020	≥ 30	Completed
NCT04542993	Zinc Picolinate; Resveratrol; Zinc Picolinate Placebo; Resveratrol Placebo	Reduction in SARS-CoV-2 Viral load; Reduction in Severity of COVID-19 Disease.	Mar 2021	60	SC	Sep 2020	Jun 2022	18-75	Active, not recruiting
NCT04468139	Quercetin; bromelain; Zinc; Vitamin C	Days of stay at hospital after treatment and discharge; serum zinc before and after treatment.	Jul 2020	60	T	Jun 2020	Jul 2020	≥ 18 years	Recruiting
NCT04377646	Hydroxychloroquine; Hydroxychloroquine (placebo); Zinc; Zinc (Placebo)	SARS CoV2 infection;	May 2020	660	P	May 2020	Jul 2020	18-65	Not yet recruiting
NCT04447534	Chloroquine; Zinc	Number of patients with improvement or mortality.	Dec 2020	200	T	Jun 2020	Oct 2030	≥18	Recruiting
NCT04351490	Zinc gluconate; 25-OH cholecalciferol	Survival rate in asymptomatic subjects at inclusion	Mar 2021	0	T	Apr 2020	Jul 2020	60	Withdrawn (change study design and funding)
NCT04342728	Ascorbic Acid; Zinc Gluconate; Ascorbic Acid and Zinc Gluconate; Standard of Care.	Symptom Reduction.	Feb 2021	214	SC	Apr 2020	Feb 2021	≥ 18	Completed
NCT04472585	Ivermectin Injectable Solution; Injectable Placebo; Zinc; Placebo empty capsule; Oral Ivermectin.	qRT-PCR; Time taken for alleviation of symptoms; Severity of symptoms.	Feb 2021	180	T	Nov 2020	Oct 2021	18-60	Recruiting
NCT04877509	Biological: Selenium, Zinc and Copper, Vitamin A, D, E plasma concentrations during patient hospitalization	Selenium, Zinc and Copper, Vitamin A, D and E plasma concentrations of 229 over 50 year's patients hospitalized suffering from COVID-19.	May 2021	229	O	Mar 2020	May 2021	50	Completed
NCT04407572	Serum zinc, vitamin d vitamin b12 levels.	Serum zinc, vitamin d vitamin b12 deficiency levels	Jun 2020	44	P	Apr 2020	Jun 2020	18-45	Completed
NCT04584567	Doxycyclin	Decreasing the number of cases infected with covid 19	Feb 2021	1100	P	Nov 2020	Mar 2021	18-65	Recruiting

NCT04395768	Vitamin C; Hydroxychloroquine; Azithromycin; Zinc Citrate Vitamin D3; Vitamin B12.	Symptoms; Length of hospital stay; invasive mechanical ventilation or mortality	Sep 2020	200	T	Sep 2020	Dec 2021	≥ 18 years	Recruiting
NCT04621461	Zinc Sulfate 220 MG; Placebo	Number of participants hospitalized and/or requiring repeat emergency room visits; Number of participants admitted to the Intensive care unit; Number of participants on a ventilator.	Feb 2021	3	T	Dec 2020	Feb 2021	30	Completed
NCT04780061	Vitamin D3 50,000 IU; Vitamin C/Zinc; Vitamin K2/D; Microcrystalline Cellulose Capsule; Medium Chain Triglyceride Oil	Participant-reported overall health	Mar 2021	200	T	Apr 2021	Dec 2021	≥18	Not yet recruiting
NCT04335084	Hydroxychloroquine; Vitamin C; Vitamin D; Zinc	Prevention of COVID-19 symptoms as recorded in a daily diary; Safety as determined by presence or absence of Adverse Events and Serious Adverse Events	Feb 2021	600	P	Jun 2020	Dec 2021	18	Recruiting
NCT04551339	PreserVision AREDS; Multivitamin with zinc	COVID-19 illness requiring hospitalization	Mar 2021	4500	O	Sep 2020	Sep 2021	18-90	Active, not recruiting
NCT04446104	Hydroxychloroquine Sulfate Tablets; Ivermectin 3mg Tab; Zinc; Povidone-Iodine; Vitamin C.	Laboratory-confirmed COVID-19 in treatment arms (hydroxychloroquine, ivermectin, zinc and povidone iodine)	Oct 2020	4257	P	May 2020	Aug 2020	21-60	Completed
NCT04392427	Combination of Nitazoxanide, Ribavirin and Ivermectin for a duration of seven days.	Negative test result for COVID-19	Sep 2020	100	T	Oct 2020	May 2022	12	Not yet recruiting
NCT04798677	ABBC1 Immunoessential; Placebo	Change in the acute immune response to the influenza vaccine after supplementation; Change in the delayed immune response to the influenza vaccine after supplementation; Change in the acute immune response to the Covid-19 vaccine after supplementation; Change in the delayed immune response to the Covid-19 vaccine after supplementation; Change in blood levels of selenium and zinc.	Mar 2021	90	O	Oct 2020	Apr 2021	18	Recruiting
NCT04326725	Plaquenil	Protection against COVID-19	Jul 2020	80	P	Mar 2020	Sep 2020	20-90	Active, not recruiting
NCT04435587	Ivermectin Pill; Combined ART/hydroxychloroquine	Adverse event rates; Efficacy for shortening duration of SAR-CoV2 detection by PCR	Jun 2021	80	T	Jul 2020	Nov 2021	18	Recruiting
NCT04528927	HCQ; Azithromycin; Doxycycline; Zinc	Evaluate the rate of patients cured at the end of the study; Evaluate the rate of patients are pauci-symptomatic at the end of the study.	Aug 2020	0	T	May 2020	Jul 2020	18-80	Withdrawn (Interest in the use of HCQ is controversi al.)
NCT04631536	Atorvastatin + L-arginine + Folic acid + Nicorandil + Nebivolol; Placebo	Clinical Improvement	Mar 2021	80	T	Jan 2021	Jun 2021	18	Recruiting
NCT04334512	Hydroxychloroquine; Azithromycin; Vitamin C; Vitamin D; Zinc	The rate of recovery of mild or moderate COVID-19 in patients using Quintuple Therapy; Reduction or Progression of Symptomatic Days; Assess the safety of Quintuple Therapy; Assess the safety of Quintuple Therapy via pulse; Assess the safety of Quintuple Therapy via oxygen saturation; Assess the safety of Quintuple Therapy via EKG; Assess Tolerability of Quintuple Therapy	Apr 2021	600	T	Jun 2020	Sep 2021	18	Recruiting

NCT04668469	Ivermectin; Hydroxychloroquine; personal protective Measures.	Number of participants with improvement of clinical condition (symptoms and signs); Reduction of recovery time, hospital stay days and mortality rate	Dec 2020	600	T	Jun 2020	Oct 2020	18-80	Completed
NCT04482686	Ivermectin; Doxycycline Hcl; Zinc; Vitamin D3; Vitamin C	Time to Non-Infectivity by RT-PCR; Time to Symptom progression in days as measured by NEWS scoring system (National Early Warning Score); Time to Symptom improvement as measured by NEWS scoring system (National Early Warning Score); Efficacy of Treatment as measured by Titer; Efficacy of Treatment as measured by RT-PCR	May 2021	30	T	Dec 2020	Jul 2022	18-75	Recruiting
NCT04810728	Extract Psidii guava; Standard therapy for Covid-19 patient	Changes of inflammatory cells and marker	Apr 2021	90	T	Jun 2020	Jan 2021	13-59	Completed
NCT04384458	Hydroxychloroquine; Ivermectin	Proportion of participants in whom there was a positivity for SARS-CoV-2.	Oct 2020	400	P	Jul 2020	Apr 2021	18-70	Recruiting
NCT04554979	Hydroxychloroquine Pill	Non-communicable disease and COVID-19; Seasonal change and COVID-19; Possible region-specific classification for COVID-19 disease.	Sep 2020	199	R	Jun 2020	Jul 2020	18	Completed
NCT04590274	Hydroxychloroquine; Vitamins and Minerals; Azithromycin	Percentage of individuals who develop COVID-19 symptoms	Oct 2020	5000	P	Nov 2020	Dec 2021	All ages	Not yet recruiting
NCT04666116	Dietary supplementation	Viral load during the period of admission to the nasopharyngeal smear.	Dec 2020	96	T	Apr 2020	Feb 2021	18-99	Recruiting
NCT04621149	Chlorine dioxide; zinc acetate; Famotidine; placebo; lactoferrin, green tea extract	Reduction in Participant Symptoms of COVID-19	Dec 2020	120	T	Nov 2020	Mar 2021	20-70	Recruiting
NCT04485130	Disulfiram; Placebo	Immunologic impact of 5 days of disulfiram, as measured by the fold-change in plasma levels of pro-inflammatory cytokines (e.g., interleukin 6, interleukin 1-beta, etc.).	Dec 2020	60	T	May 2020	Apr 2021	18	Recruiting
NCT04507867	Nutritional support system; Control	Complete clinical and dietary history, complete blood count, blood chemistry, D-dimer, coagulation times, bleeding time and prothrombin time, fibrinogen, serum electrolytes, serum homocysteine levels, serum levels of 25 hydroxycholecalciferol, triceps fold and arm circumference, % muscle mass, % fat, BMI, weight, height.	Aug 2020	240	SC	Aug 2020	Dec 2020	30-75	Not yet recruiting
NCT04371952	Doxycycline, placebo	Percentage of Patients with Clinical Respiratory Aggravation; Percentage of patients hospitalized; Percentage of patients requiring ventilatory assistance	May 2020	330	T	Jun 2020	Dec 2020	> 45	Not yet recruiting
NCT04523831	Ivermectin and Doxycycline; Standard of care	Number of Patients with Early Clinical Improvement; Number of Participants With Late Clinical Recovery.	Oct 2020	400	T	Jun 2020	Sep 2020	18	Completed
NCT04751669	Vitamin and trace elements; Placebo	Need for hospital admission	Apr 2021	300	T	Apr 2021	Dec 2021	18-80	Not yet recruiting
NCT04666753	Immuno Formulation	Clinical symptoms duration.	Dec 2020	40	R	Jul 2020	Sep 2020	18	Completed
NCT04777981	CBDRA60 supplement; Placebo.	Decreased hospitalization.	Mar 2021	60	T	Apr 2021	May 2021	18	Not yet recruiting
NCT04323228	Oral supplement enriched in antioxidants; cellulose-containing placebo	Score of Nutrition risk screening-2002 (NRS); Serum ferritin level; Serum Interleukin-6; Serum C-reactive protein concentration; Serum Tumor necrosis factor-α concentration; Serum monocyte chemoattractant protein 1.	Sep 2020	40	SC	Sep 2020	Dec 2020	18-65	Recruiting

NCT04491994	HCQ	Number of Participants with Progression	Aug 2020	540	T	Apr 2020	May 2020	18-80	Completed
NCT04591600	Ivermectin and Doxycyline; Standard of care	Mortality; Rate of progression disease;	Oct 2020	140	T	Jul 2020	Oct 2020	16-86	Completed
NCT04485169	Therapeutic Plasma Exchange	Survival;	Sep 2020	280	T	Apr 2020	Jul 2020	18-80	Completed
NCT04537962	Colgate Periogard® mouthwash; Colgate Peroxyl® mouthwash; Colgate Total® Mouthwash; Toothpaste with sodium monofluorophosphate; Toothpaste with sodium fluoride and zinc; Toothpaste with tin fluoride.	Reduction of SARS-CoV-2 load in the oral mucosa and saliva	May 2021	202	SC	Jun 2020	Aug 2021	18-90	Enrolling by invitation
NCT04542213	Linagliptin tablet; Insulin	Glucose levels; Number of patients who achieve metabolic control;	Mar 2021	70	T	Aug 2020	Feb 2021	18-75	Completed
NCT04532931	Standard of care (Paracetamol); Artesunate-amodiaquine; Pyronaridine-artesunate; Favipiravir plus Nitazoxanide; Sofosbuvir/daclatasvir	Incidence of SARS-CoV-2 clearance	Dec 2020	250	T	Sep 2020	Oct 2021	18-65	Recruiting

Footnote: The studies were selected from the U.S. National Library of Medicine (assessed at ClinicalTrials.gov) by the descriptors “COVID-19 | Melatonin | Vitamin C | Zinc”. Abbreviations: ***n***, Estimated Enrollment of participants; ***Prim. Purp***., Primary Purpose; ***Estim.Compl. Date*****:** Estimated Completion Date; **NT**: not recruiting.
